# Repeated Bouts of Advanced Strength Training Techniques: Effects on Volume Load, Metabolic Responses, and Muscle Activation in Trained Individuals

**DOI:** 10.3390/sports7010014

**Published:** 2019-01-06

**Authors:** William Wallace, Carlos Ugrinowitsch, Matt Stefan, Jacob Rauch, Christopher Barakat, Kevin Shields, Andrew Barninger, Renato Barroso, Eduardo O. De Souza

**Affiliations:** 1 Department of Health Science and Human Performance, University of Tampa, Tampa, FL 33606, USA; williamwallace603@gmail.com (W.W.); mstefan2423@gmail.com (M.S.); jacobrauch1@gmail.com (J.R.); chris@competitivebreed.com (C.B.); kevinshields31@gmail.com (K.S.); abarninger@gmail.com (A.B.); 2 Leadership—Health and Human Performance Department, Concordia University Chicago, River Forrest, IL 60305, USA; 3 Laboratory of Adaptations to Strength Training, School of Physical Education and Sport, University of Sao Paulo, São Paulo 05508-030, Brazil; ugrinowitsch@gmail.com; 4 School of Physical Education, University of Campinas, Campinas 13083-851, Brazil; barroso@fef.unicamp.br

**Keywords:** resistance training, advanced training techniques, metabolic stress, muscle swelling

## Abstract

This study investigated the effects of advanced training techniques (ATT) on muscular responses and if performing a second training session would negatively affect the training stimulus. Eleven strength-trained males performed a traditional strength training session (TST) and four different ATT: pre-exhaustion A (PE-A), pre-exhaustion B (PE-B), forced repetitions (FR), and super-set (SS). On day 1, SS produced lower volume load than TST, FR, and PE-B (−16.0%, *p* ≤ 0.03; −14.9, *p* ≤ 0.03 and −18.2%, *p* ≤ 0.01, respectively). On day 2, SS produced lower volumes than all the other ATT (−9.73–−18.5%, *p* ≤ 0.03). Additionally, subjects demonstrated lower perceived exertion on day 1 compared to day 2 (6.5 ± 0.4 AU vs. 8.7 ± 0.3 AU, *p* = 0.0001). For blood lactate concentration [La-] on days 1 and 2, [La-] after the tenth set was the highest compared to all other time points (baseline: 1.7 ± 0.2, fifth-set: 8.7 ± 1.0, tenth-set 9.7 ± 0.9, post-5 min: 8.7 ± 0.7 mmol∙L^−1^, *p* ≤ 0.0001). Acute muscle swelling was greater immediately and 30-min post compared to baseline (*p* ≤ 0.0001). On day 2, electromyography (EMG) amplitude on the clavicular head of the pectoralis major was lower for SS than TST, PE-A, and PE-B (−11.7%, *p* ≤ 0.01; −14.4%, *p* ≤ 0.009; −20.9%, *p* = 0.0003, respectively). Detrimental effects to the training stimulus were not observed when ATT (besides SS) are repeated. Strength trained individuals can sustain performance, compared to TST, when they are using ATT in an acute fashion. Although ATT have traditionally been used as a means to optimize metabolic stress, volume load, and neuromuscular responses, our data did not project differences in these variables compared to TST. However, it is important to note that different ATT might produce slight changes in volume load, muscle excitation, and fluid accumulation in strength-trained individuals from session to session.

## 1. Introduction

It is widely accepted that strength training (ST) is an effective tool for enhancing functional and morphological adaptations in skeletal muscles (i.e., muscle strength and hypertrophy, respectively) [1]. Regarding the ST stimulus, it has been proposed that increments in training volume as well as muscle activation, are two of the most effective training parameters [2,3,4,5] to induce adaptations in skeletal muscle. Accordingly, research has demonstrated that high training volumes resulted in greater muscle hypertrophy in strength-trained individuals [6]. Furthermore, muscle activation can be assessed by surface electromyography (EMG) [7]. It has been suggested that the greater the amplitude of the EMG signal the higher the muscle activation, which is positively associated with muscle strength production [7]. Alternatively, some authors have suggested that high ST-induced metabolic stress may also play a role (i.e., fluid accumulation, lactate, inorganic phosphate, etc.) in inducing strength training adaptations [8,9]. Thus, trained individuals should implement ST strategies capable of optimizing the aforementioned variables (i.e., training volume, motor unit recruitment, and metabolic stress).

It has been suggested that advanced training techniques (ATT) may properly manipulate training volume, muscle activation, and metabolic stress in order to maximize strength training-induced adaptations [10]. The most frequent ATT used by practitioners and strength coaches are supersets, forced repetitions, and pre-exhaustion. There is evidence which suggests higher metabolic stress accumulation (i.e., blood lactate and creatine kinase) in a superset training session (i.e., two different exercises sets consecutively followed by a recovery period) compared to a traditional ST (TST) session [11,12]. Similarly, forced repetitions (FR), which are characterized by a training partner assisting the lifter after concentric failure just enough to keep moving the weight through the range of motion for additional repetitions, produce greater session volume load, muscle fatigue, and muscle activation compared to TST [13]. Furthermore, the pre-exhaustion (PE) technique is characterized by performing a single joint exercise to fatigue the synergist muscle group or the agonist muscles, before performing a multi-joint exercise. This is typically done in an attempt to increase muscle activation in the prime movers of a multi-joint exercise. However, existing evidence on strength-trained individuals utilizing PE techniques ensuing greater training load, muscle activation, and metabolic stress is limited [14,15].

To the best of our knowledge, no study has attempted to determine the residual fatigue induced by training sessions using these ATT. For instance, one could expect that the ATT producing the highest metabolic stress would hamper total volume, muscle activation, and possibly metabolic stress in the following training session. Consequently, assessing the residual effects of the superset, forced repetitions, and pre-exhaustion training sessions would help practitioners and strength coaches organize training sessions within a microcycle more effectively. Therefore, the purpose of this study was two-fold: (1) to determine which ATT maximize volume load, muscle activation, swelling and blood lactate concentration [La-] in strength-trained individuals and (2) to determine if performing a second strength training session 48 h later would enhance performance or negatively affect the training stimulus on the subsequent session. We hypothesized that different ATT would demonstrate a similar pattern to TST on volume load, muscle activation, acute muscle swelling and [La-]. However, we assumed that ATT might affect the same variables in a different way from TST when they are repeated within 48 h.

## 2. Materials and Methods

### 2.1. Participants

Eleven strength-trained college males (age: 24 ± 4 years, height: 171 ± 4 cm, body mass: 88 ± 23 kg, ST experience: 6 ± 5 years, bench-press 10 repetitions maximum [10 RM]: 106 ± 42 kg) volunteered for this study. Inclusion criteria consisted of having a minimum of one year of ST experience and the ability to perform the bench press exercise with a load corresponding to 1.2 times their body mass as being able to bench press greater than 1.2 times their body mass objectively demonstrates their training status. All subjects were free of any functional or medical limitations that would preclude them to perform the experimental protocol. Subjects were excluded if they reported any use of ergogenic aids or anabolic steroids. All subjects were informed of the purposes, procedures, benefits, and risks due to study participation and prior to the commencement of the study. All the subjects read and signed an informed consent approved by the Institutional Review Board.

### 2.2. Familiarization

All the participants performed two familiarization sessions interspersed by a minimum of 48 h prior to the commencement of the study to determine the flat and incline bench press exercises 10 repetition maximum (10 RM) load, which were used in all experimental sessions. During familiarization sessions, subjects performed a general warm-up consisting of a five minute run on a treadmill at 8 km·h^−1^. Afterward, they performed a specific warm-up consisting of 10 repetitions at 50% of estimated 1 RM followed by five repetitions at 70% of 1 RM. A 2-min rest interval was allowed between the two sets. Tests for 10 RM in the flat and incline bench press were performed in a counterbalanced and randomized order on each familiarization session (i.e., one exercise per familiarization session). As these were strength trained individuals, they performed two sets, on each familiarization session, to obtain the 10 RM load on each exercise. Participants were given five-minute rest intervals between 10 RM attempts. For PE-A, subjects’ 10 RM for a triceps pushdown exercise was also determined.

### 2.3. Experimental Sessions

Participants underwent five different training sessions: (Traditional [TST], Forced Repetition [FR], Superset [SS], Pre-exhaustion A [PE-A] and Pre-exhaustion B [PE-B]). The order subjects performed each of the experimental sessions was balanced and randomized following William’s square design. In addition, each technique was performed twice per week interspersed by 48 h. The subject’s 10 RM load was used for the first set of all the conditions as follows: TST—subjects performed 5 × 10 RM on the flat bench-press, followed by 5 × 10 RM on the incline bench-press. FR—participant performed both flat bench-press and incline exercises following an 8 RM to failure, plus two forced repetitions with the assistance from the same spotter for a total of 10 repetitions per set. SS—consisted of 5 × 10 RM on flat bench-press immediately followed by 5 × 10 RM on an incline bench with no rest between exercises, and a 120 s rest period between sets. PE-A—initially, participants performed 5 × 10 RM of a triceps pushdown cable machine exercise, followed by 5 × 10 RM on flat bench-press and 5 × 10 RM on the incline bench-press. PE-B—participants switched the order of exercises in which they began with the incline bench-press (i.e., subjects performed 5 × 10 RM on the incline bench-press, followed by 5 × 10 RM on the flat bench-press). In the event that a participant was able to complete more than 10 repetitions on a single set, an instruction was given to end the set at 10 repetitions. Otherwise, all sets were completed to concentric failure (i.e., the inability to complete concentric phase of the lift with proper form), whether failure occurred at 10 repetitions or less. The load was adjusted on each proceeding set to ensure that participants reached failure as closest to 10 repetitions (8 for FR) as possible for each technique. 120 s and 180 s of rest were granted between sets and exercises, respectively. Strong verbal encouragement was given for all participants, regardless of the ATT used. Participants were allowed to train their lower body muscles during the experimental protocol, but refrain from training their upper body muscles. Additionally, participants were instructed to maintain their dietary habits throughout the study.

### 2.4. Volume Load and Perceptual Assessments

Volume load (kg) was calculated from individual experimental sessions logs for each participant utilizing the formula: total repetitions (reps) × weight (kg).

Upon entering the laboratory on experimental sessions, participants were asked to sit down and determine their perceived recovery (i.e., 0–10 scale [PRS]) on that given day as follows: zero and ten indicate very poorly recovered/extremely tired and very well recovered/highly energetic, respectively [16]. The rate of perceived exertion (RPE) was assessed immediately after and 30 min following the experimental session. RPE measurement was based on the CR-10 RPE scale. Instructions and procedures for using the PRS and RPE scale were given to all individuals during the familiarization sessions. In addition, all assessments were performed in isolation from other subjects to ensure accuracy.

### 2.5. Metabolic Stress

Ultrasonography (GE LOGIQ^®^; General Electric Company, Fairfield, CT, USA) was used to determine the acute fluid accumulation (i.e., acute muscle swelling) of the pectoralis major muscle at the sternal (MSS) and clavicular (MSC) heads, using a linear array probe with a frequency of 8.0 MHz. Muscle swelling was assessed at the location that EMG electrodes were fixed (described below). To obtain the images, subjects laid supine with their legs fully extended and their muscles relaxed. A water-soluble gel was applied to the transducer to aid acoustic coupling and eliminate pressure deformation of the tissues underneath the probe. Scans were performed on the right side of the body with the transducer perpendicularly oriented to the sternoclavicular joint. Ultrasound settings were kept consistent for each testing session. Additionally, Permanent markers were used to mark and measure the same designated spot on each participant. Every participant was given a permanent marker and asked to maintain the site of measurement throughout the course of the study. The same-blinded investigator performed all of the ultrasound assessments. Acute muscle swelling measures were acquired at rest, immediately and 30-min post experimental sessions. The coefficient of variation (CV) was determined prior to the study using five different participants with similar characteristics to the current participants. The CV for acute muscle swelling assessments was ~2.0%. Muscle swelling assessments were performed at the same time of the day, on three different days interspersed by 48 h. 

Blood lactate concentration [La-] was assessed using a lactate analyzer (Lactate Plus, Nova Biomedical, Waltham, MA, USA). After sterilizing the finger, a puncture was made with a spring-loaded single use disposable lancet. The first drop of blood was discarded and the second was applied to an assay strip and inserted into the lactate analyzer. La—samples were taken at baseline, after the fifth set, at the rest interval between exercises (5th-set), immediately after the tenth set (10th-set), and post-5 min experimental session, except in the case of SS where the mid measurement for [La-] was taken after the third set (i.e., after six sets combined). This marked the closest time point to the middle measurement (i.e., between exercises), relative to the other techniques.

### 2.6. Overall Session EMG

Muscle activation (EMG) was recorded using a 16-channel electromyography system (Trigno, Delsys, Boston, MA, USA) with an acquisition frequency of 2000 Hz and a hardware band-pass filter of 20–450 Hz. The skin area was shaved, abraded, and cleaned with an isopropyl alcohol pad to reduce skin impedance before electrode placement. Two active bar electrodes (10 mm center-to-center) were positioned on the midclavicular line, midway between the acromioclavicular joint and the sternoclavicular joint, over the second (i.e., sternocostal head of the pectoralis major) and fifth intercostal (i.e., sternal portion of the pectoralis major) spaces, respectively [17,18], parallel to the presumed orientation of the muscle fibers. The position of each electrode during the first session was marked on the skin with a semi-permanent marker, and marks were reapplied on each successive visit to the laboratory. A maximum bench-press isometric contraction was performed before each experimental session. The participants were positioned lying on the bench with the shoulder and the elbow at a 90° angle. Participants were instructed to perform a 3 s ramp contraction followed by an immediate 5-s maximum voluntary contraction (MVIC). Two total MVICs were performed, with 3 min given between MVICs. We determined maximal muscle activation during the MIVC selecting a 500 ms window in which EMG values were maximal. In addition, as there is high between day variations in EMG, this procedure was performed on each experimental session, therefore our EMG was expressed as the percentage of the maximal activation on each experimental session. The raw electromyography signals were digitally filtered (4th order Butterworth, band pass 20–500 Hz) and converted to root mean square (RMS). For the dynamic contractions, RMS was calculated for the entire set and normalized by the highest values obtained during the isometric contraction in 500 ms windows. Average EMG data was calculated for the entire session for each muscle portion (i.e., overall session EMG).

### 2.7. Statistical Analysis

After normality (i.e., Shapiro Wilks) and variance assurance (i.e., Levene), a mixed model was performed for each dependent variable (e.g., volume load, PRS, RPE, muscle swelling, La- and EMG). As the experimental design has up to three fixed factors (i.e., condition, day and time) and differing levels on each factor, different mixed models were used. For example, volume load, EMG, PRS, and RPE was analyzed using a mixed model having condition (e.g., TST, FR, SS, PE-A and PE-B) and day (e.g., 1 and 2) as a fixed factor, and subjects as a random factor. For acute muscle swelling and La, a mixed model was used assuming ATT (e.g., TST, FR, SS, PE-A and PE-B), day (e.g., 1 and 2) and time (e.g., acute muscle swelling: baseline, immediately post and 30-min post for muscle swelling and La-: baseline, 5th-set, 10th-set and post-5 min) and subjects as random factor (SAS 9.4, SAS Institute Inc., Cary, NC, USA). Whenever a significant F-value was obtained, a post-hoc test with a Tukey’s adjustment was performed for multiple comparison purposes [19]. The significance level was previously set at *p* < 0.05 with a trend toward significance being accepted at *p* ≤ 0.10. For selected variables, within and between group effect sizes (ES) were calculated as follows: [(M2-M1)/standard deviation pooled] and we have presented the 95% confidence intervals (95% CI). Results are expressed as mean ± standard deviation (SD). 

## 3. Results

### 3.1. Volume Load and Perceptual Assessments

On day 1, there were significant differences in volume load between ATT (*p* = 0.01). SS produced lower volume load than TST, FR, and PE-B (−16.0%, ES: −1.15; −14.9%, ES: −1.05, and −18.2%, ES: 1.37, respectively). On day 2, SS produced lower volume load than all the other techniques (−9.73% to −18.5%, ES: 0.90–1.83), (*p* = 0.0001). In addition, PE-B and FR produced greater volume load than PE-A (*p* = 0.01), (8.3%, ES: 0.74; 10.7%, ES: 0.92, respectively) (Table 1). In addition, there was a certain trend towards significance (*p* = 0.08) greater volume loads on day 2 when compared to day 1 (2.3%, ES: 0.34).

For RPE, there was a main effect for day (*p* = 0.001), in which subjects demonstrated lower perceived exertion levels on day 2 than day 1 (6.5 ± 0.4 AU vs. 8.7 ± 0.3 AU, ES: 5.6). For PRS, there were no significant differences across techniques and days (*p* ≥ 0.05), (Table 2).

### 3.2. Metabolic Stress

For the acute muscle swelling of the sternocostal (MSS) and clavicular (MSC) heads, there was a significant main time effect (*p* = 0.0001) on day 1 in which acute muscle swelling was significantly greater immediately and 30 min post experimental sessions when compared to baseline (*p* = 0.0001). In addition, acute muscle swelling immediately post was greater than 30-min post experimental sessions (*p* = 0.0001). On day 2, similar results were observed as MSS and MSC were significantly greater immediately and 30 min post experimental sessions when compared to baseline (*p* = 0.0001). Similarly, muscle swelling immediately post was greater than 30-min post experimental sessions (*p* = 0.0001), (Table 3 and Table 4).

**Figure 1:**
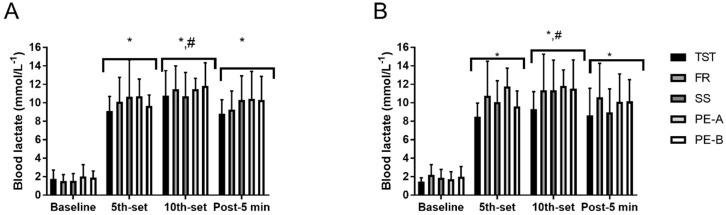
Whole body lactate concentration. (**A**)—day 1; (**B**)—day 2. * *p* ≤ 0.05 significantly greater than baseline; # *p* ≤ 0.05 significantly greater fifth-set and post-5 min.

For [La-], there was a main effect for time on day 1 (*p* = 0.0001) in which all conditions significantly increased [La-] in similar fashion when compared to baseline. In addition, [La-] after tenth set demonstrated the highest value when compared to other time points (baseline: 1.7 ± 0.1, fifth-set: 8.7 ± 0.6, tenth-set: 9.8 ± 0.4, post-5 min: 8.8 ± 0.7 mmol∙L^−1^), (*p* = 0.0001). On day 2, similar results were observed as [La-] significantly increased in similar fashion when compared to baseline. Similar to day 1, [La-] at post demonstrated the highest value when compared to other time points on day 2 (baseline: 1.7 ± 0.2, 5th-set: 8.7 ± 1.0, 10th-set 9.7 ± 0.9, post-5 min: 8.7 ± 0.7 mmol∙L^−1^), (*p* = 0.0001). Furthermore, there was a strong trend to toward significant main effect of technique (*p* = 0.0506), in which [La-] tended to be higher in PE-A when compared to TST (PE-A: 8.8 ± 4.1 = mmol∙L^−1^, TST: 6.9 ± 3.1 mmol∙L^−1^), (Figure 1).

### 3.3. Overall Session EMG

**Figure 2:**
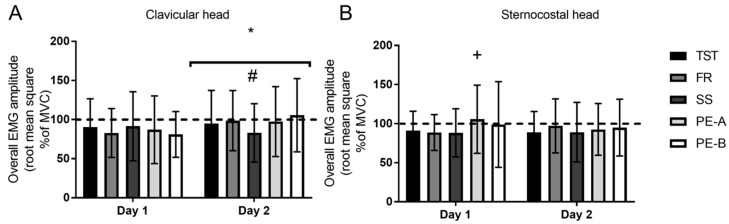
Overall session surface electromyography (root mean square % of maximum isometric voluntary contraction). (**A**)—Clavicular head; (**B**)—Sternocostal head. * *p* ≤ 0.05 significantly greater than day 1; # *p* ≤ 0.05 significantly lower than TST, PE-A, and PE-B, + *p* ≤ 0.05 significantly greater PE-B and SS.

For the clavicular head, there was a trend toward a significant main effect for technique (*p* = 0.07) on day 1, in which overall EMG amplitude (i.e., RMS for all sets combined) tended to be higher in TST when compared to FR (9.7%, *p* = 0.07). On day 2, there was a main effect for technique (*p* = 0.0002), in which overall EMG amplitude was lower for SS when compared to TST, PE-A, and PE-B systems (−11.7%, *p* = 0.01; −14.4%, *p* = 0.009; −20.9%, *p* = 0.0003, respectively). Furthermore, there was a significant main day effect (*p* = 0.0001), in which overall EMG amplitude was greater on day 2 when compared to day 1 (10.6%, *p* = 0.0001), (Figure 2A).

For the sternocostal head, there was a main effect for technique (*p* = 0.01) on day 1 in which PE-A demonstrated greater overall EMG amplitude when compared to PE-B and SS (7.1%, *p* = 0.008; 19.3%, *p* = 0.03, respectively). In addition, there was a trend towards significantly greater EMG amplitude for PE-A compared to TST (15.3%, *p* = 0.09). On day 2, there were no significant differences between conditions (*p* > 0.05). In addition, PE-A demonstrated a trend to decrease overall EMG on day 2 when compared to day 1 (−11.4%, *p* = 0.07), (Figure 2B).

## 4. Discussion

Our main findings indicate that the superset decreased volume load and muscle activation compared to the other ATT and traditional strength training. The effect size data suggest that TR, FR, and PE-B techniques produced a greater increase in acute muscle swelling (Table 2). Interestingly, strength-trained individuals demonstrated lower perceived exertion on day two compared to day one.

### 4.1. Volume Load and Perceptual Measures

On day one, superset produced lower volume than traditional strength training, forced-repetition, and pre-exhaustion-B (i.e., incline bench-press followed by flat bench-press) techniques. In addition, superset demonstrated the lowest volume load compared to all other techniques. (e.g., day 1: −9.7%, day 2: −18.5%, *p* = 0.03) on day two. Our findings contradict previous research suggesting that the superset technique can be used without any detrimental effect on volume load [20,21]. However, comparison between these studies should be taken with caution. Most of the previous literature refers to agonist-antagonist, or alternating upper and lower body superset techniques [12,20,21,22], whereas we have utilized superset technique for the same muscle group. Furthermore, our data suggest that compared to the traditional strength training session, none of the tested ATT affected volume loads. Soares et al. [14] had subjects perform 1 × 10 RM using a pre-exhaustion technique similar to PE-A (i.e., synergistic exercise performed prior the core-compound exercise) used in the current study. These researchers demonstrated that pre-exhaustion and traditional strength training sessions produced similar volume loads in strength-trained individuals, which coincide with our findings. Moreover, an important goal of the current study was to assess whether an ATT would either enhance or decrease volume load on the subsequent training session. Interestingly, our subjects were able to perform similar volume loads on day one and two (e.g., day 1: 6189.6 ± 386.1 kg, day 2: 6332.6 ± 447.8 kg, 2.3%). Therefore, strength-trained individuals performing a second ATT session 48 h later did not produce a negative effect on the subsequent training session.

In regards to perceptual assessments, our results expand the findings of previous research demonstrating that traditional strength training and pre-exhaustion sessions affect levels of perceived exertion in strength-trained individuals in a similar fashion [14]. However, Balsamo et al. [23] suggested that reductions in training session duration (i.e., increasing work to rest ratio [kg∙min^−1^]) might result in increased RPE. Additionally, Weakley et al. [12] found that RPE was significantly higher for volume matched superset and tri-sets than it was for TST, as the time to complete the sessions was lower. Our findings do not corroborate with previous research. Superset produced similar RPE to the other techniques despite participants producing significantly less volume load and taking less time to complete the experimental session. Furthermore, participants reported significantly lower RPE on day 2. Considering that RPE is affected by both physiological and psychological factors [24], this reduction in RPE may be the result of: (a) an improvement in their readiness, suggesting that the subjects were more mentally prepared to manage the stimulus on the second training session or (b) they learned how to pace themselves better throughout the second training session.

### 4.2. Metabolic Stress

Acute muscle swelling and blood lactate concentrations were used as a marker of metabolic stress in the current study. Regarding acute muscle swelling, we reported an average ~13.5% increase immediately after training for days 1 and 2. Our data are in agreement with previous research that demonstrated ~15.0% increase in pectorals major muscle swelling in strength-trained individuals, performing different modes of chest-press exercises in acute fashion [25]. However, our effect sizes suggest that traditional strength training, force-repetition and pre-exhaustion-B sessions had greater magnitude of fluid accumulation compared to other techniques (Table 2). Although subjects in force-repetition had an external assistance to complete the last two repetitions of each set, forced-repetition, traditional strength training and pre-exhaustion-B were the techniques that produced the greatest volume loads. This increase in volume may have placed a greater overload on the chest muscles, which may explain the greater responses in acute muscle swelling observed in these three techniques.

Interestingly, our acute muscle swelling findings do not concur with [La-] results. However, our findings are in agreement with current literature demonstrating that strength-trained individuals had higher [La-] in response to high-volume strength training sessions [12,26]. In addition, the [La-] response was similar amongst conditions, despite superset utilizing shorter rest over the course of 10 sets between the flat bench press and the incline bench press. Weakley et al. [12] demonstrated that volume equated SS and tri-set techniques resulted in significantly more [La-] accumulation over the course of 18 sets than a traditional strength training session. In addition, Kelleher et al. [11] found that a work matched superset techniques produced larger [La-] than a traditional strength training session. However, the former study employed a protocol where similar muscle groups were not worked consecutively. In the case of the latter, agonist-antagonist were used; whereas, our superset technique utilized the same muscle group between exercises. Despite lower overall volume, it is likely that the shorter rest intervals and less overall rest allowed for similar [La-] to be produced in the superset technique employed in the current study.

### 4.3. Overall Session EMG

Regarding muscle activation, our findings demonstrated that the superset technique produced less clavicular head EMG amplitude than traditional strength training, and pre-exhaustion A and B sessions. Likewise, previous research showed decreases in EMG activity when one chest exercise is immediately followed by another [15]. The higher metabolic stress (relative to time), in the superset technique may attenuate neural drive to the muscles reducing muscle activation [27].

In addition, the force-repetition technique used in this study is thought to increase muscle activation in strength-trained individuals and subsequently induce larger amounts of neuromuscular fatigue [7,28]. Ahtiainen et al. [13] demonstrated that forced-repetition resulted in significantly less EMG amplitude of the vastus lateralis and vastus medialis during leg press, squats, and leg extensions, when compared to traditional strength training session. However, our findings do not completely coincide with those aforementioned studies, as EMG amplitude only tended to be higher in both heads of pectoralis major during traditional strength training session when compared to force-repetition on day 1. Nonetheless, despite relative intensity being higher during the force-repetition technique (i.e., subjects performed their 8 RM, with additional assistance provided to complete the 10 reps), similar muscle activation was observed across conditions. As volume load was not different between forced-repetition and the other techniques (besides superset), it is possible that in strength-trained individuals, different training techniques might result in similar levels of muscle activation in order to achieve similar volume loads.

In regards to the pre-exhaustion techniques, our findings corroborate with previous studies that did not find differences in pectoralis major activation comparing traditional strength training and pre-exhaustion sessions in trained individuals [14,29]. However, pre-exhaustion-A showed an increase in pectoralis activation compared to pre-exhaustion-B and superset. While, it is difficult to compare these findings to current literature due to lack of available data on the effects of different ATT on muscle activation. Our findings suggest that performing a compound movement prior to performing the flat bench-press exercise and/or utilizing superset for the same muscle group might decrease pectoralis activation compared to pre-exhaustion-A technique (i.e., performing single joint exercise for accessory muscle groups prior the bench-press) in trained individuals. Still, it is important to point out that there were no differences in pectoralis activation across conditions in sternocostal head on day 2.

### 4.4. Limitations

Our study has several inherent limitations. (1) We did not measure muscle swelling 24 h post training. Therefore, we cannot paint an accurate picture of the time course of fluid accumulation for each individual. (2) We did not measure muscle activity of any other muscles besides the clavicular and sternal heads of pectoralis major. By including measurements of the triceps brachii or anterior deltoid we may have been able to draw better comparisons between studies. Most of the current literature has assessed muscle activation of synergists and prime movers of the upper body during different strength training techniques that include the bench press exercise. (3) Our population consisted of young strength-trained males. Therefore, we cannot generalize our findings for older trained participants (i.e., older than 30 years) nor trained female individuals. (4) Although the participants were strictly instructed to maintain their training regimens and refrain from exercise 48 h before each experimental session, there is no way for us to say with entire confidence that these individuals did not perform any co-founding factors prior to an experimental session. Finally, this was an acute study. Further research is needed to examine the chronic effects of perceived fatigue and actual performance variables when repeatedly using different ATT in strength-trained individuals.

## 5. Conclusions

In conclusion, the superset technique employed herein produced the lowest volume load and induced more neuromuscular fatigue compared to the other ATT, despite similar responses in metabolic stress and perceptual assessments. In addition, pre-exhaustion-A produced greater muscle excitation than pre-exhaustion-B and superset in the sternocostal head. However, pre-exhaustion-A demonstrated a trend to decrease overall muscle excitation when this technique was repeated in the following session. Regarding metabolic stress, effect sizes suggest that traditional strength training, force-repetition and pre-exhaustion-B sessions had greater fluid accumulation (i.e., acute muscle swelling) compared to other techniques. On the contrary, [La-] responses were similar across ATT and traditional strength training sessions. Perhaps, the most interesting finding of the current manuscript is that strength-trained individuals are able to tolerate ATT in a similar fashion to traditional strength training. The ATT did not produce negative performance measures and residual fatigue besides superset. Although ATT have traditionally been used as a means to optimize metabolic stress, volume load and neuromuscular responses, our data did not project differences in these variables compared to traditional strength training. However, it is important to note that different advanced techniques might produce slight changes in volume load, muscle excitation, and fluid accumulation in strength-trained individuals from session to session.

## Figures and Tables

**Table 1:** Volume load (kg). Data are mean (±SD).

Conditions	Day 1	Day 2	95% CI	ES	P Day	P Condition	P Condition by Day
TST	6466 ± 957	6474 ± 957	−780 kg–728 kg	0.00	0.07	0.002	0.73
FR	6408 ± 941	6815 ± 738	−762 kg–755 kg	0.48			
SS	5573 ± 604 *	5554 ± 630 ^+^	−1133 kg–319 kg	0.03			
PE-A	5907 ± 583	6153.1 ± 686	−1015 kg–496 kg	0.38			
PE-B	6591 ± 859	6666 ± 687	−855.4–656 kg	0.09			

TST—Traditional strength training, FR—Forced repetition, SS—superset, PE-A—Pre-exhaustion A, PE-B—Pre-exhaustion B. 95% CI—Within day 95% confidence intervals, ES—Within day effect size, * *p* ≤ 0.05 significantly lower than TST, FR, and PE-B; ^+^ *p* ≤ 0.05 significantly lower than all the other techniques.

**Table 2:** Rate of perceived exertion (RPE) and perceived recovery scale (PRS). Data are mean (±SD).

**RPE (A.U)**							
**Conditions**	**Day 1**	Day 2 *	**95% CI**	**ES**	**P Day**	**P Condition**	**P Condition by Day**
TST	9.1 ± 1.1	5.9 ± 1.9	−5.1–−1.1	−2.5	0.01	0.95	0.19
FR	8.9 ± 1.1	6.7 ± 2.4	−4.1–−0.2	−1.7			
SS	8.5 ± 1.5	6.3 ± 1.5	−4.1–−0.2	−1.7			
PE-A	8.9 ± 1.1	6.4 ± 1.4	−4.5–−0.4	−1.9			
PE-B	8.3 ± 1.4	7.3 ± 2.1	−1.0–3.0	−0.7			
**PRS**							
**Conditions**	**Day 1**	**Day 2**	**95% CI**	**ES**	**P Day**	**P Condition**	**P Condition by Day**
TST	6.9 ± 1.1	7.1 ± 0.9	−1.5–1.0	0.2	0.66	0.07	0.91
FR	7.8± 1.4	7.9 ± 1.3	−1.3–1.1	0.1			
SS	7.5 ± 0.6	7.8 ± 1.4	−1.4–1.0	0.3			
PE-A	7.3 ± 0.9	7.10 ± 0.7	−1.0–1.5	−0.2			
PE-B	7.2 ± 1.0	7.2 ± 0.9	−1.2–1.3	0.0			

TST—Traditional strength training, FR—Forced repetition, SS—superset, PE-A—Pre-exhaustion A, PE-B—Pre-exhaustion B. 95% CI—Within day 95% confidence intervals, ES—Within day effect size, * *p* ≤ 0.05 significantly lower than day 1.

**Table 3:** Acute muscle swelling of the sternocostal head (cm). Data are mean (±SD).

**Day 1**									
**Conditions**	**Baseline**	**Post**	**Post-30 min**	**ES (Pre-Post)**	**ES (Pre-30 min-Post)**	**Condition**	**Day**	**Time**	**Condition × Time × Day**
TST	2.08 ± 0.44	2.49 ± 0.50 *^,#^	2.29 ± 0.46 *	1.10	0.56	0.69	0.26	0.0001	0.87
FR	2.05 ± 0.33	2.39 ± 0.32 *^,#^	2.20 ± 0.30 *	0.91	0.40				
SS	2.07 ± 0.41	2.43 ± 0.42 *^,#^	2.25 ± 0.35 *	0.96	0.48				
PE-A	2.24 ± 0.29	2.49 ± 0.50 *^,#^	2.25 ± 0.49*	0.67	0.66				
PE-B	1.98 ± 0.38	2.49 ± 0.46 *^,#^	2.26 ± 0.44 *	1.36	0.74				
**Day 2**									
TST	2.16 ± 0.35	2.54 ± 0.44 *^,#^	2.35 ± 0.39 *	1.22	0.47				
FR	2.05 ± 0.35	2.42 ± 0.32 *^,#^	2.24 ± 0.29 *	0.92	0.47				
SS	2.07 ± 0.43	2.34 ± 0.45 *^,#^	2.14 ± 0.36 *	0.67	0.17				
PE-A	2.14 ± 0.46	2.44 ± 0.51 *^,#^	2.28 ± 0.42 *	0.75	0.35				
PE-B	2.03 ± 0.39	2.44 ± 0.40 *^,#^	2.30 ± 0.41 *	1.02	0.67				

* *p* ≤ 0.05 significantly greater than baseline; ^#^ *p* ≤ 0.05 significantly greater than post-30 min.

**Table 4:** Acute muscle swelling of the clavicular head (cm). Data are mean (±SD).

**Day 1**									
**Conditions**	**Baseline**	**Post**	**Post-30 min**	**ES (Pre-Post)**	**ES (Pre-30 min-Post)**	**Condition**	**Day**	**Time**	**Condition × Time × Day**
TST	1.99 ± 0.27	2.21 ± 0.35 *^,#^	2.16 ± 0.30 *	0.43	0.33	0.61	0.65	0.0001	0.96
FR	2.07 ± 0.32	2.37 ± 0.34 *^,#^	2.19 ± 0.36 *	0.59	0.23				
SS	2.27 ± 0.95	2.31 ± 0.33 *^,#^	2.18 ± 0.31 *	0.07	−0.17				
PE-A	2.19 ± 0.28	2.23 ± 0.31 *^,#^	2.14 ± 0.31 *	0.07	−0.09				
PE-B	1.98 ± 0.33	2.32 ± 0.31 *^,#^	2.18 ± 0.41 *	0.67	0.39				
**Day 2**									
TST	2.10 ± 0.31	2.26 ± 0.23 *^,#^	2.18 ± 0.27 *	0.55	0.27				
FR	2.08 ± 0.29	2.33 ± 0.26 *^,#^	2.19 ± 0.28 *	0.86	0.38				
SS	2.06 ± 0.31	2.25 ± 0.32 *^,#^	2.08 ± 0.36 *	0.65	0.06				
PE-A	2.19 ± 0.21	2.28 ± 0.30 *^,#^	2.11 ± 0.30 *	0.31	0.27				
PE-B	2.01 ± 0.31	2.35 ± 0.40 *^,#^	2.21 ± 0.40 *	1.17	0.69				

* *p* ≤ 0.05 significantly greater than baseline; ^#^ *p* ≤ 0.05 significantly greater than post-30 min.
